# Editorial: Identification and functional analysis of differentially expressed genes in plant response to abiotic stresses

**DOI:** 10.3389/fpls.2023.1246964

**Published:** 2023-07-20

**Authors:** Xuke Lu, Libei Li, Yupeng Cui, Ting Zhao, Waqar Afzal Malik

**Affiliations:** ^1^ Institute of Cotton Research of Chinese Academy of Agricultural Sciences/National Engineering Research Center of Cotton Biology Breeding and Industrial Technology/Zhengzhou Research Base, National Key Laboratory of Cotton Bio-breeding and Integrated Utilization, School of Agricultural Sciences, Zhengzhou University, Anyang, Henan, China; ^2^ College of Advanced Agricultural Sciences, Zhejiang A&F University, Hangzhou, China; ^3^ School of Biology and Food Engineering, Anyang Institute of Technology, Anyang, China; ^4^ College of Agriculture & Biotechnology, Zhejiang University, Hangzhou, China; ^5^ Chinese Academy of Agricultural Sciences (CAAS), Beijing, China

**Keywords:** abiotic stress, differentially expressed genes, response, mechanism, function analysis

## Overview

As the impact of climate change intensifies, plants face increasing challenges in adapting to a rapidly changing environment. Abiotic stresses, such as drought, salinity, extreme temperatures, and nutrient deficiency, pose significant threats to crop productivity and global food security. It has been reported that more than 8% of the world’s agricultural land was salinized, and the area of salt-affected land is predicted to double by 2050 (Rengasamy, 2006; Guan et al., 2014). In response to these stresses, plants have evolved intricate molecular mechanisms to sense and respond to environmental cues, thereby enhancing their survival and productivity. The identification and functional analysis of differentially expressed genes (DEGs) in plant responses to abiotic stresses has become an essential area of research, aiming to unravel the underlying genetic basis of stress tolerance and facilitate the development of resilient crop varieties (Zhang et al., 2022).

Our Research Topic titled “*Identification and Functional Analysis of Differentially Expressed Genes in Plant Response to Abiotic Stresses*” (40012) was published on 22 July 22, 2022, and completed on 12 February, 2023. A total of 62 authors confirmed the e-mails, and finally 35 manuscripts were successfully submitted, among which 23 manuscripts were accepted for publication. In this topic, numerous differentially expressed genes (DEGS) were identified and functionally analyzed in many types of plants, such as maize, sunflower, cotton, soybean, rice, *Solanum lycopersicum* L., Salix matsudana, Passiflora edulis Sims, wheat, sweet potatoes, Panax ginseng, Santalum album, sorghum, and Populus, providing more valuable information for molecular mechanism research of plants in responding to abiotic stresses.

## Unveiling the transcriptomic landscape

In recent years, advances in high-throughput sequencing technologies, such as RNA sequencing (RNA-seq), have revolutionized our ability to comprehensively characterize the plant transcriptome. RNA-seq enables the simultaneous quantification of gene expression levels across the entire genome, providing an unbiased and precise snapshot of gene activity under specific stress conditions. Through comparative transcriptomic analyses between stressed and control plants, researchers have successfully identified numerous DEGs involved in stress response pathways. These findings have offered crucial insights into the molecular networks governing plant adaptation to abiotic stresses.

### Functional analysis: connecting genes to stress response pathways

Identifying DEGs is just the first step towards understanding their functional roles in the plant stress response. Functional analysis, including gene ontology (GO) enrichment, pathway analysis, and gene regulatory network reconstruction, plays a pivotal role in deciphering the intricate mechanisms underlying stress tolerance. GO enrichment analysis reveals the biological processes, molecular functions, and cellular components associated with the identified DEGs, highlighting the key biological pathways involved in stress adaptation. Pathway analysis provides a holistic view of the interconnected metabolic and signaling pathways orchestrating stress responses, aiding in the identification of critical genes and regulatory hubs. Additionally, gene regulatory network reconstruction elucidates the intricate interactions and hierarchical relationships among stress-responsive genes, offering a systems-level understanding of stress adaptation.

### Integrating omics approaches: towards a systems biology perspective

The complexity of abiotic stress responses necessitates the integration of multiple omics approaches to achieve a comprehensive understanding. Transcriptomics, proteomics, metabolomics, and epi-genomics collectively provide a multi-dimensional view of the stress response, unraveling the interplay between genes, proteins, metabolites, and epigenetic modifications. Integration of these omics datasets allows researchers to identify key regulatory nodes, discover novel stress-responsive molecules, and gain insights into the cross-talk between different layers of regulation. By adopting a systems biology perspective, we can uncover emergent properties and regulatory modules that would remain elusive when studying individual components in isolation.

## Research progress

Corn is the oil and feed crop with the largest sown area in China, and it plays an important role in agricultural production (Mousavi et al., 2022). High seed vigor and germination rates are crucial for corn production for its potential in high quality and yield of crops. Jin et al. performed whole-genome-wide identification of miRNAs and their targets associated with maize seed vigor, finally a total of 791 mature miRNAs were gained, among of which 286 miRNAs were newly identified to be closely related with seed vigor. Ding et al. identified 129 maize NAC protein-coding genes, of which 15 and 20 NAC genes were differentially expressed between the two genotypes H082183 and Lv28 based on transcriptome analysis. Jasmonic acid (JA), one of the Phytohormones, is always involved in modulating physiological and molecular responses to abiotic stresses (Wolters and Jurgens, 2009; Garrido-Bigotes et al., 2019). Song et al. conducted the phylogenetic analysis and expression profiles of 139 putative JAZ genes, and the results indicated that JAZ genes in sunflower were conserved in sequence but varied in their expression among duplicated HaJAZ genes, suggesting that these JAZ genes may confer neo-functionalization in the responses to abiotic stresses. Environment factors, such as temperature, drought, salt, sunshine duration, significantly influenced oil and FA compositions in soybean, thus Zuo et al. identified QTN-by-environment (QEIs) interactions and their candidate genes for soybean seed oil-related traits using 3VmrMLM method, providing important information for genetic basis, molecular mechanisms, and soybean breeding. In rice, two density of direct seeding with high and normal density were selected by Cui et al. to identify the differentially expressed genes (DEGS) involved in shade-avoidance syndrome. The results indicated that simulation of shade environment could cause rapid decrease in the expression of phytochrome genes, expression changes of multiple miR156 or miR172 genes and photoperiod-related genes. Normal growth and development of tomatoes (*Solanum lycopersicum* L.) as well as fruit quality and yield are limited by salt stress, so Wang et al. revealed the transcriptional regulatory network of hormones and genes under salt stress in tomato plants.

Phenotypes of *Salix matsudana* females and males are obviously different under salinity stress, and the molecular mechanisms were decoded by Liu et al. based on the root transcriptome of males and females. Yang et al. conducted genome-wide characterization and identification of Trihelix transcription factors and expression profiling in response to abiotic stresses in Chinese Willow (*Salix matsudana* Koidz) to reveal the molecular mechanism when responding to various abiotic stresses. Wheat (*Triticum aestivum* L.) is one of the world’s most widely cultivated crops (Alghabari et al., 2015). At present, no documents about chromosome condensation 1 (RCC1) domain proteins have been found. An et al. performed genome-wide identification and expression analysis of the regulator of RCC1 gene family in wheat. MYB TFs, common regulators of gene transcription, form a large gene family involved in a variety of biological processes in plants (Dai et al., 2023). Identification of the passion fruit (*Passiflora edulis* Sims) MYB family in fruit development and abiotic stress was performed by Zhang et al., laying the foundation for further analysis of the biological functions of PeMYBs involved in stress resistance in passion fruit. Tang et al. identified 144 R2R3-MYB genes and investigated their characteristics and expression patterns in *Santalum album*. Sweet potato, regarded as the seventh most important food crop, is a popular food source in many countries, i.e., China, India, and Kenya. Liu et al. revealed the molecular mechanism of two sweet potatoes with different drought tolerances using transcriptome analysis. Tong et al. also used transcriptome analysis to obtain 672 upregulated genes and 526 downregulated genes in the roots of rusty ginseng when compared with the healthy ginseng roots. Jiao et al. revealed the regulation mechanism of the flavonoid biosynthesis pathway in responding to cadmium stress by integration analysis of transcriptome and metabolome in sorghum. Guo et al. identified a total of 30 heat shock transcription factor (HSF) members in poplar, and overexpression of *PsnHSF21* conferred salt tolerance in *Populus simonii × P. nigra* by functional analysis.

Cotton is the main raw material of textile industry in China and as well as the world in general, and it plays a pivotal role in our national economy (Lu et al., 2019). In this topic, a total of eight articles related to cotton tolerance were published. Yan et al. reported that *GhCDPK60* positively regulated drought stress tolerance in both transgenic *Arabidopsis* and cotton by regulating proline content and ROS level. The *GhSRS21* gene was proved to negatively control cotton salt tolerance by regulating the balance between ROS production and scavenging by Sun et al. Documents demonstrated that subtilisin-like proteases (SBTs) are a large family of serine peptidases that are unique to plants and are closely associated with environmental responses (Page and Di Cera, 2008; Jin et al., 2021). Dai et al. identified 120 and 112 SBTs in the tetraploid cotton species *G. hirsutum* and *G. barbadense*, while 67 and 69 SBTs were identified in the diploid species *G. arboreum* and *G. raimondii*, respectively. In several plant species, NITRATE TRANSPORTER 1/PEPTIDE TRANSPORTER (NPF) genes are reported to play crucial roles in plant growth, development and resistance to various stresses, involving in the transport of nitrate (NO3-) and peptides (O’brien et al., 2016; Fan et al., 2017). Liu et al. identified 201 genes encoding NPF proteins with a peptide transporter (PTR) domain in three different Gossypium species, and investigated expression profiles of the NPF genes. Cui et al. identified a total of 260 Terpene synthases (TPS) in four cotton species and conducted their expression profiles for their crucial characteristics in response to flooding stress. Trihelix transcription factors (TTFs) play important roles in abiotic stress responses in many plant species. Li et al. reported that the overexpression of the cotton trihelix transcription factor *GhGT23* in *Arabidopsis* could mediate salt and drought stress tolerance by binding to GT and MYB promoter elements in stress-related genes. In addition, *GhSS9* and *GhLDAP2* were reported to enhance drought tolerance of cotton by Dai et al. and Zhao et al., respectively.

## Future perspectives

In summary, the work published in this Research Topic showed many differentially expressed genes (DEGs) and transcription factors were identified and functionally analyzed in responding salinity, drought, temperatures, and waterlogging stress. As we all know, plant tolerance is a complex quantitative trait, involving a series of DEGs and networks. Although several important research progresses were published in topic, it is far from enough to elucidate how plant tolerances are formed. Based on this Research Topic, it is very necessary to carry out further research into the following issues: 1) manuscripts related to proteomics, miRNAs, DNA methylation, and gene editing should be encouraged; 2) regulation networks of multiple genes should be constructed; 3) interaction mechanism of stress-related genes; 4) the influence of environmental factors on subcellular structures in plants.

## Author contributions

XL conceived and wrote the paper. LL, YC, TZ and W.M contributed to the article arrangement and revision. All authors listed have made a substantial, direct, and intellectual contribution to the work and approved it for publication.
